# Activation of Metabisulfite by Dissolved Fe(III) at Environmentally Relevant Concentrations for Organic Contaminants Degradation

**DOI:** 10.3390/ijms26030953

**Published:** 2025-01-23

**Authors:** Jianan Chen, Longjiong Chen, Leliang Wu, Chengyu Yan, Ningxin Sun, Guilong Peng, Shaogui Yang, Huan He, Chengdu Qi

**Affiliations:** 1 School of Environment, Jiangsu Province Engineering Research Center of Environmental Risk Prevention and Emergency Response Technology, Jiangsu Engineering Lab of Water and Soil Eco–remediation, Nanjing Normal University, Nanjing 210023, China; 2 State Key Laboratory of Resource Insects, College of Sericulture, Textile and Biomass Sciences, Southwest University, Chongqing 400715, China; 3 Hubei Key Laboratory of Mineral Resources Processing and Environment, Wuhan University of Technology, Wuhan 430070, China

**Keywords:** metabisulfite, ferric ion, organic contaminants, sulfate radical, degradation mechanism

## Abstract

Currently, iron-catalyzed low-valent sulfur species processes are regarded as potentially valuable advanced oxidation processes (AOPs) in wastewater treatment. As a commonly used low-valent sulfur species in the food industry, metabisulfite (MBS) can undergo decomposition to bisulfite when dissolved in water. Therefore, the combination of MBS with dissolved Fe(III) at environmentally relevant concentrations is proposed in this study to accelerate organic contaminants degradation while simultaneously minimizing the production of iron sludge. The results show that the Fe(III)/MBS process could degrade various organic contaminants, including acid orange 7 (AO7), and the removal efficiency of AO7 obeyed the pseudo-first-order kinetic. The rate constant values exhibited variations depending on the initial concentrations of Fe(III) and MBS, pH values, as well as the reaction temperature. Moreover, the contribution of HO^•^ and SO_4_^•−^ to AO7 degradation was estimated as 51.59% and 46.45%, respectively. Furthermore, Cl^−^ showed a minimal effect while HCO_3_^−^ and humic acid resulted in a significant inhibitory effect on AO7 degradation. The satisfactory degradation of AO7 was achieved in three real water bodies. Ultimately, the results of gas chromatography–mass spectrometry and the theoretical calculations greatly facilitate the proposal of AO7 degradation pathways, including N=N cleavage, hydroxylation, and hydrogen abstraction. The findings of this study indicate that the Fe(III)/MBS process may be a promising AOP for further application in organic contaminants degradation during wastewater treatment.

## 1. Introduction

Since 2003, there has been growing interest in the use of sulfate radical-based advanced oxidation processes (AOPs) for the removal of organic contaminants in wastewater treatment [1]. So far, persulfates (peroxymonosulfate (PMS) and peroxydisulfate (PDS)) are the most frequently used sulfate radical precursors, although persulfates have the disadvantages of costliness and chronic toxicity [2]. Thus, low-valent sulfur species, such as (bi)sulfite, thiosulfate, and dithionite, with less toxicity and competitive cost were considered promising persulfate substitutes [3]. Among the various activation methods for low-valent sulfur species, transition metal is one of the possibilities for sulfate radical formation [4]. Furthermore, iron-activated low-valent sulfur species processes present advantages, including their economical and environmentally friendly characteristics [5]. At the current stage, iron-mediated low-valent sulfur species processes have been applied to organic contaminants destruction in wastewater treatment successfully [4,6].

Compared to other common low-valent sulfur species sources ((bi)sulfite, thiosulfate, and dithionite), metabisulfite (MBS, S_2_O_5_^2−^) can be dissociated into twofold bisulfite (HSO_3_^−^) when dissolved in water (Table S1) [7] and has the advantage of acidic pH, which obviates the need for pH adjustment [8]. In addition, MBS is often used as an antioxidant to quench residual chlorine in water treatment and preservative agents in the food industry [9]. Furthermore, MBS has been used directly in wastewater treatment to convert highly toxic Cr(VI) to less toxic Cr(III) [10,11]. Recently, heterogeneous iron-containing-material-activated MBS processes have been found to be capable of degrading organic contaminants [7,12,13,14]. Considering that iron plays an important role in modern wastewater treatment in general, in which it is used to prevent hydrogen sulfide emissions during anaerobic digestion and acts as a coagulant to improve sludge dewatering [15], and significant amounts of dissolved iron (typically: 0.5–1.5 mg/L) have been found in wastewater [15], the dissolved Fe(III)-activated MBS process may have the potential to degrade concurrent organic contaminants, which reflects the idea of treating waste with waste. Additionally, we found that dissolved Fe(III)-activated MBS for organic contaminants degradation, especially in the case of dissolved Fe(III) at environmentally relevant concentrations, is rarely studied.

Therefore, we proposed that dissolved Fe(III) at environmentally relevant concentrations activated the MBS process to focus on studying the performance and mechanism of organic contaminants removal. The specific objectives of this study are to (1) explore the feasibility of implementing dissolved Fe(III) at environmentally relevant concentrations for MBS activation and its application for the degradation of organic contaminants, including acid orange 7, which is a representative azo dye with “carcinogenic, teratogenic and mutagenic” effects [16]; (2) investigate the influences of several important parameters, including the initial Fe(III), MBS concentration, pH value, reaction temperature, as well as water matrix on the degradation efficiency of AO7; (3) ascertain the dominant reactive oxygen species generated in the Fe(III)/MBS process; and (4) propose a potential AO7 transformation pathway.

## 2. Results and Discussion

### 2.1. Removal of Organic Contaminants in the Iron-Activated Metabisulfite Process

**Figure 1:**
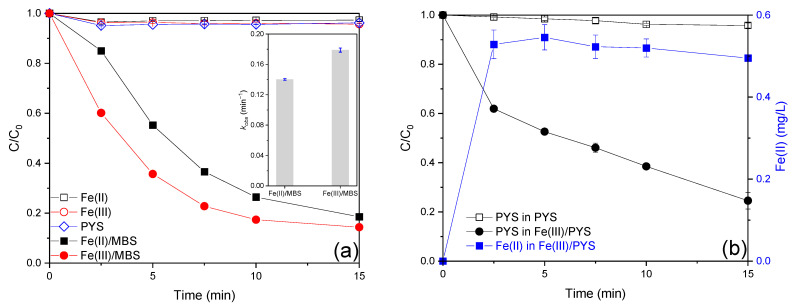
(**a**) Degradation efficiency of AO7 in the iron ion-activated MBS processes and (**b**) changes of MBS and Fe(II) concentration in the Fe(III)/MBS process (reaction condition: [AO7]_0_ = 0.02 mM, [Fe(II)]_0_ = [Fe(III)]_0_ = 0.01 mM, [MBS]_0_ = 0.1 mM, T = 25 °C).

The AO7 removal efficiency in the presence of dissolved iron ions at environmentally relevant concentrations (Fe(II) or Fe(III), 0.01 mM, 0.56 mg/L) was less than 5.0% (Figure 1a), suggesting the possibility of complex formation between AO7 and iron ions (Figure S1) [17]. When MBS was introduced, only 3.8% of AO7 was decolorized (Figure 1a), indicating the weak oxidation ability of MBS toward AO7 [18]. However, 81.5% and 85.6% of AO7 degradation efficiency can be obtained in the Fe(II)/MBS and Fe(III)/MBS processes (Figure 1a), respectively, manifesting the synergistic effect between MBS and iron ion for AO7 degradation [19]. In addition, AO7 removal in the iron/MBS processes followed the pseudo-first-order kinetic, with the rate constant values (*k_obs_*) of 0.140 and 0.179 min^−1^ for Fe(II)/MBS and Fe(III)/MBS processes (Figure 1a), respectively. The enhanced efficiency of the Fe(III)/MBS process compared with the Fe(II)/MBS process may be attributed to the internal electron transfer that occurs within the Fe(III)–sulfite complex. This mechanism necessitates the involvement of O_2_, facilitating oxidation into Fe(III)–sulfite, which subsequently undergoes internal electron transfer [6]. The AO7 degradation performance observed in the present study is comparable to the results previously reported for the Fe(III) catalyzed S(IV) auto-oxidation process in the context of organic contaminant decontamination ([5,19,20,21,22,23,24,25,26,27], Table S2). In addition, the concentration changes of MBS and Fe(II) in the Fe(III)/MBS process were monitored. As shown in Figure 1b, the consumption of MBS in the MBS process was merely 4.2%, indicating the negligible auto-oxidation ability of MBS. However, 75.4% of MBS was depleted and around 0.5 mg/L Fe(II) was generated within 15 min in the Fe(III)/MBS process. These results demonstrated that the Fe(III)/MBS process exhibited a high capacity for the consumption of MBS and Fe(II) generation.

Furthermore, the performance of AO7 degradation in the Fe(III)/peroxygen (H_2_O_2_, PMS, and PDS) processes was examined. As exhibited in Figure S2, the decolorization efficiency of AO7 in the Fe(III)/peroxygen processes was less than 5%, indicating that the ability of Fe(III) to mediate the decomposition of the above three oxidants to generate reactive species is negligible [17]. Moreover, the Fe(III)/MBS process exhibited the best removal of AO7 compared with the Fe(III)/HSO_3_^−^ process (83.2%) and Fe(III)/SO_3_^2−^ process (8.5%), which may be attributed to the differing pH levels of the solutions employed. The MBS solution was acidic, the sulfite solution was alkaline, and the bisulfite solution had a weakly acidic pH [19].

Moreover, the Fe(III)/MBS process was also employed for the degradation of a range of common aquatic organic contaminants, including dyes (rhodamine B and methyl orange), phenolic compounds (phenol and acetaminophen), and pharmaceuticals (tetracycline and sulfamethoxazole). As shown in Figure S3, the Fe(III)/MBS process achieved a satisfactory degradation efficiency (>70.0%) for all the tested aquatic organic contaminants, implying the nonselective reactive species generated in the Fe(III)/MBS process. It is notable that the effluent contained 0.56 mg/L of iron, which meets the municipal wastewater emission standard set by several countries. This indicates that the effluent can be directly emitted into natural waters without the necessity for post-treatment of dissolved iron [28]. The aforementioned results indicated that the Fe(III)/MBS process is a promising AOP that can be used to remove a wide range of aquatic organic contaminants.

### 2.2. Effect of Reaction Parameters

**Figure 2:**
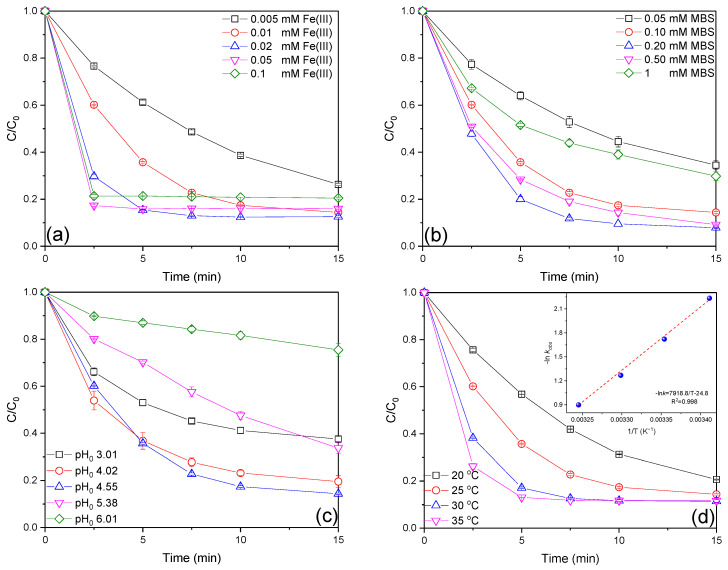
Effect of initial (**a**) Fe(III), (**b**) MBS concentration, (**c**) solution pH, and (**d**) reaction temperature on AO7 degradation efficiencies in the Fe(III)/MBS process (except for the investigated parameter, the other parameters were fixed at [AO7]_0_ = 0.02 mM, [Fe(III)]_0_ = 0.01 mM, [MBS]_0_ = 0.1 mM, T = 25 °C).

As a critical factor in the Fe(III)/MBS process, the impact of the initial Fe(III) dosage on AO7 degradation in the Fe(III)/MBS process was examined. As illustrated in Figure 2a, the AO7 removal efficiencies at 0.005, 0.01, 0.02, 0.05, and 0.10 mM Fe(III) were 73.7%, 85.6%, 87.4%, 84.0%, and 79.5%, respectively. The overall AO7 removal efficiency demonstrated an increase with the elevated Fe(III) dosage from 0.005 to 0.02 mM while the sustained elevation of the Fe(III) dose to 0.10 mM resulted in a decline in efficacy. Additionally, the increase in Fe(III) concentration from 0.005 to 0.05 mM significantly accelerated the *k_obs_* values from 0.0889 to 0.702 min^−1^ (Figure S4a), which may be attributed to the constant generation of reactive species generated at the given MBS dose, and the fact the excess Fe(III) can compete with AO7 for reactive species at higher initial Fe(III) concentrations [17]. Furthermore, the formation of Fe(OH)_3_ colloids at elevated Fe(III) concentrations may also hinder AO7 removal [20].

Subsequently, the effect of the initial MBS concentration on AO7 degradation in the Fe(III)/MBS process was investigated. As seen in Figure 2b, the AO7 degradation efficiencies at 0.05, 0.10, 0.20, 0.50, and 1 mM MBS were 65.5%, 85.6%, 92.1%, 90.8%, and 70.2%, respectively. The overall AO7 removal efficiency increased as MBS concentration rose from 0.05 to 0.20 mM but showed a decline at higher concentrations. This reduction in removal efficiency at elevated MBS concentrations can be attributed to the quenching or scavenging of reactive species by excess MBS, thereby reducing the availability of reactive species for AO7 removal. The *k_obs_* values followed a similar trend, confirming this relationship (Figure S4b).

Solution pH plays a pivotal role in the removal efficacy of organic contaminant degradation in AOPs. The speciation of Fe(III) and S(IV) is significantly influenced by pH, which in turn regulates the concentration of catalytically active Fe–S(IV) complexes [29]. As shown in Figure 2c, the AO7 removal efficiencies at initial solution pH 3.01, 4.02, 4.55, 5.38, and 6.01 in the Fe(III)/MBS process were 62.4%, 80.5%, 85.6%, 66.3%, and 24.5%, respectively. The overall AO7 removal efficiency demonstrated an initial increase as pH increased from 3.01 to 4.55, followed by a subsequent decline when pH was further elevated to 6.01. As illustrated in Figure S5, the solution pH exhibited minimal fluctuations when the initial pH was 3.01. Conversely, an initial decline in pH was observed when the initial pH ranged from 4.02 to 6.01, ultimately stabilizing at a new pH level. Furthermore, it was observed that the final solution pH remained within a narrow pH range of 3.5–4.0 despite the initial pH exhibiting a considerable variation between 4.02 and 5.38. The changes in *k_obs_* values are consistent with that of the overall AO7 removal efficiency (Figure S4c). The observed pH-dependent degradation of AO7 can be attributed to the combined effect of pH on the distribution of MBS species and iron speciation. MBS has two pKa values (1.90 and 7.20) [30], resulting in the formation of different concentrations of SO_2_·H_2_O, HSO_3_^−^, and SO_3_^2−^ at varying pH values (Figure S6). It was therefore established that the predominant species of MBS was SO_3_^2−^ under alkaline conditions, while HSO_3_^−^ was the most significant species in the acidic pH region. Furthermore, sulfite was observed to decline and convert to SO_2_·H_2_O at an extremely acidic pH (pH < 3). Furthermore, Fe(III) will rapidly undergo hydrolysis and precipitation in the form of ferric oxyhydroxide at a higher pH [31], thereby reducing the concentration of dissolved Fe(III) available for MBS activation.

The reaction temperature is another important operating parameter for the chemical reaction. As shown in Figure 2d, the removal efficiencies of AO7 at 20, 25, 30, and 35 °C were 79.3%, 85.6%, 88.6%, and 88.3%, respectively. The removal efficiency increased with raised reaction temperature, indicating that AO7 removal in the Fe(III)/MBS process is endothermic. Additionally, the *k_obs_* values also increased from 0.107 to 0.407 min^−1^ with increasing reaction temperature (Figure S4d), which was attributed to the positive impacts of thermal treatment on MBS activation by Fe(III). Moreover, the raised temperature might also enhance the collision between reactive species and AO7 to promote degradation. The Arrhenius equation (Equation (1)) was employed to further fit the temperature dependency of the *k_obs_* values.

(1) $$\ln k_{obs}=\mathrm{lnA}-E_{a}/\mathrm{RT}$$

where *k_obs_* is the pseudo-first-order kinetic rate constant (min^−1^), A is the pre-exponential factor (min^−1^), E_a_ is the Arrhenius activation energy (kJ mol^−1^), R is the universal gas constant (8.314 J mol^−1^ K^−1^), and T is the absolute temperature (K).

The activation energy (E_a_) in the Fe(III)/MBS process was calculated to be 65.84 kJ mol^−1^ (insert in Figure 2d), which was close to that of the CuNSi/sulfite process (60.14 kJ mol^−1^) [32] and much lower than that of the Fe(II)/bisulfite process (120.75 kJ mol^−1^) [33], the microwave/sulfite process (353.54 kJ mol^−1^) [34], and the conventional heating-activated sulfite process (464.25 kJ mol^−1^) [34].

### 2.3. Possible Activation Mechanism

**Figure 3:**
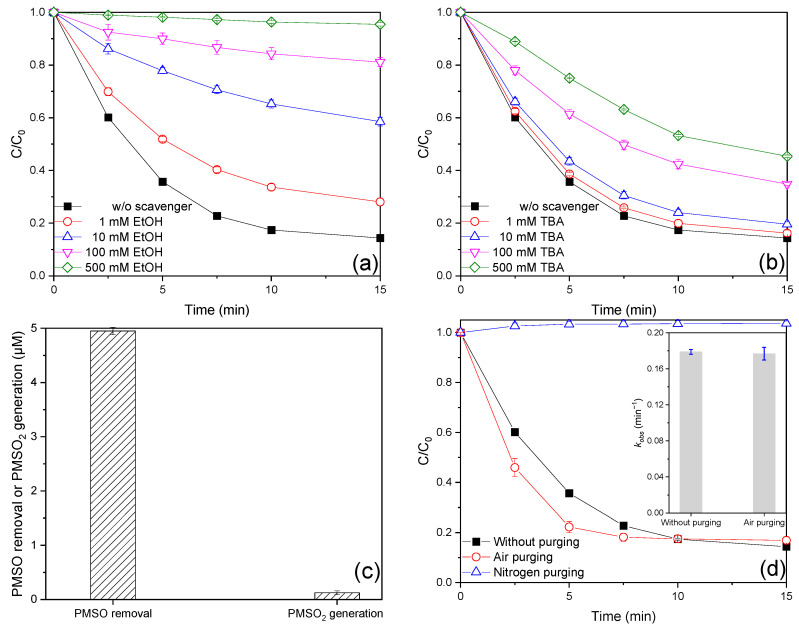
Effect of (**a**,**b**) radical scavengers and (**d**) dissolved oxygen on AO7 degradation efficiencies, and (**c**) PMSO removal, PMSO_2_ generation in the Fe(III)/MBS process (reaction condition: [AO7]_0_ = 0.02 mM or [PMSO]_0_ = 0.1 mM, [Fe(III)]_0_ = 0.01 mM, [MBS]_0_ = 0.1 mM, T = 25 °C).

It is widely accepted that SO_3_^•−^, SO_4_^•−^, SO_5_^•−^, HO^•^, and Fe(IV) are the primary reactive species involved in the iron-catalyzed S(IV) process [4,6]. The low oxidation ability of SO_3_^•−^ and its rapid reaction with oxygen (*k*_O2,SO3•−_ = 1.5 × 10^9^ M^−1^ s^−1^) result in its role in the degradation of organic pollutants being consistently overlooked in iron-activated S(IV) processes. In order to identify the reactive species involved in the Fe(III)/MBS process, quenching experiments were conducted, in which different amounts of TBA and EtOH were added, respectively. EtOH typically functions as scavenger for both HO^•^ and SO_4_^•−^, but not for SO_5_^•−^ (*k*_EtOH,HO•_ = (1.6–2.2) × 10^9^ M^−1^ s^−1^, *k*_EtOH,SO4•−_ = (1.6–7.7) × 10^7^ M^−1^ s^−1^, *k*_EtOH,SO5•−_ ≤ 10^3^ M^−1^ s^−1^). TBA exhibits scavenging activity for HO^•^, but not for SO_4_^•−^ and SO_5_^•−^ (*k*_TBA,HO•_ = (4.2–7.6) × 10^8^ M^−1^ s^−1^, *k*_TBA,SO4•−_ = (4.0–9.1) × 10^5^ M^−1^ s^−1^, *k*_TBA,SO5•−_ ≤ 10^3^ M^−1^ s^−1^). Therefore, EtOH could be employed to distinguish the contributions of SO_5_^•−^ and SO_4_^•−^/HO^•^, whereas TBA could be utilized to differentiate the contributions of SO_4_^•−^ and HO^•^. As illustrated in Figure 3a,b, the AO7 removal efficiencies were found to be diminished with varying dosages of EtOH or TBA addition. The application of EtOH and TBA at the same dosage revealed that EtOH exhibited a greater inhibitory effect on AO7 degradation than TBA, suggesting that SO_4_^•−^ and HO^•^ may be the dominant reactive species. The relative contributions of reactive species were calculated based on the difference in the *k_obs_* value (Figure S7), as detailed in Table S4. Nevertheless, the aforementioned method proved inadequate for elucidating the relative contributions of these reactive species, as a substantial excess of TBA could nonselectively scavenge SO_4_^•−^ and HO^•^ in conjunction during the Fe(III)/MBS process. Accordingly, the value of *ck* was employed to assess the competitive capacity of the scavengers for reactive species. Here, *c* represents the concentration of the scavengers, while *k* denotes the reaction rate constant of the scavengers with reactive species [21]. The rate constants of AO7 with HO^•^, SO_4_^•−^, and SO_5_^•−^ are as follows: *k*_AO7,HO•_ = 1.2 × 10^10^ M^−1^ s^−1^, *k*_AO7,SO4•−_ = 8.07 × 10^9^ M^−1^ s^−1^, *k*_AO7,SO5•−_ = 2.1 × 10^6^ M^−1^ s^−1^ [22]. The scavenging capacities of 500 mM EtOH for HO^•^, SO_4_^•−^, and SO_5_^•−^ (Table S5) were approximately 3333–4583 times, 49–239 times, and ≤12 times greater than those of 20 μM AO7 for HO^•^, SO_4_^•−^, and SO_5_^•−^, respectively. Therefore, 500 mM EtOH was sufficient to scavenge HO^•^ and SO_4_^•−^ in the Fe(III)/MBS process, but not SO_5_^•−^. The inhibition of 500 mM EtOH may be considered to represent the cumulative contribution of HO^•^ and SO_4_^•−^. Consequently, the contribution of HO^•^ and SO_4_^•−^ to AO7 degradation was calculated to be 98.04%. Correspondingly, the contribution of other species, primarily pertaining to SO_5_^•−^, was determined to be 1.96%. Given that the c*k* value of 100 mM TBA for HO^•^ was approximately 175–316 times greater than that of 20 μM AO7, 100 mM TBA was sufficient to scavenge HO^•^ in the Fe(III)/MBS process. Concurrently, the c*k* value of 20 μM AO7 for SO_4_^•−^ was observed to be 1.7–4.0 times greater than that of 100 mM TBA, thereby indicating that 100 mM TBA was unable to scavenge SO_4_^•−^ in the Fe(III)/MBS process. Therefore, 100 mM TBA may be employed to discern the relative contributions of HO^•^ and SO_4_^•−^. The estimated contributions of HO^•^ and SO_4_^•−^ to AO7 degradation were 51.59% and 46.45%, respectively. The findings indicated that SO_4_^•−^ and HO^•^ were of comparable significance in AO7 degradation within the Fe(III)/MBS process.

In order to investigate the potential occurrence of Fe(IV) in the Fe(III)/MBS process, PMSO was employed as the probe compound, given that Fe(IV) could selectively transform PMSO into a distinctive oxygen product, PMSO_2_, which differed from the radical-induced by-products [35]. As illustrated in Figure 3c, the concentrations of PMSO_2_ produced and PMSO removed were 0.125 μM and 4.95 μM within 15 min, respectively, indicating that Fe(IV) was also involved in the Fe(III)/MBS process. Furthermore, the yield of PMSO_2_, defined as the molar ratio of PMSO_2_ produced to PMSO removed, can be employed to assess the role of Fe(IV). The yield of PMSO_2_ in the Fe(III)/MBS process was found to be only 2.5%, suggesting that the effect of Fe(IV) on AO7 removal was negligible. In conclusion, the generation of Fe(IV) also occurred, although it made a minor contribution to AO7 removal in the Fe(III)/MBS process.

It is of great importance to gain an understanding of the role of DO in the activation process of S(IV), given that DO typically exerts a significant influence on the removal of organic contaminants by affecting the formation of reactive species. Therefore, the effect of DO on AO7 removal in the Fe(III)/MBS process was investigated by purging air or nitrogen, respectively. As depicted in Figure 3d, the removal efficiency and *k_obs_* value of AO7 with air purging were 83.1% and 0.177 min^−1^, respectively, which were identical to those observed in the absence of purging. Nevertheless, AO7 removal was entirely prevented through the use of purging nitrogen. These findings suggest that oxygen is a crucial factor in the transformation of AO7 in the Fe(III)/MBS process. Furthermore, the concentration of DO was monitored (Figure S8). In the absence of purging, the concentration of DO declined rapidly from 7.7 mg/L to 0 mg/L in 57 s, before increasing to reach a stable concentration of approximately 6.6 mg/L. This may be attributed to the redissolution of O_2_ under open atmospheric conditions [5]. A similar phenomenon was observed under air purging. Nevertheless, in the case of nitrogen purging, there was no significant change in DO levels which remained at approximately 0 mg/L. The DO depletion during the reaction further confirmed the involvement of O_2_ in the Fe(III)/MBS process. This may be attributed to the combined contribution of Fe(II) oxidation and the rapid reaction between SO_3_^•−^ and O_2_ to produce SO_5_^•−^ [19,23].

### 2.4. Effect of Water Matrix

**Figure 4:**
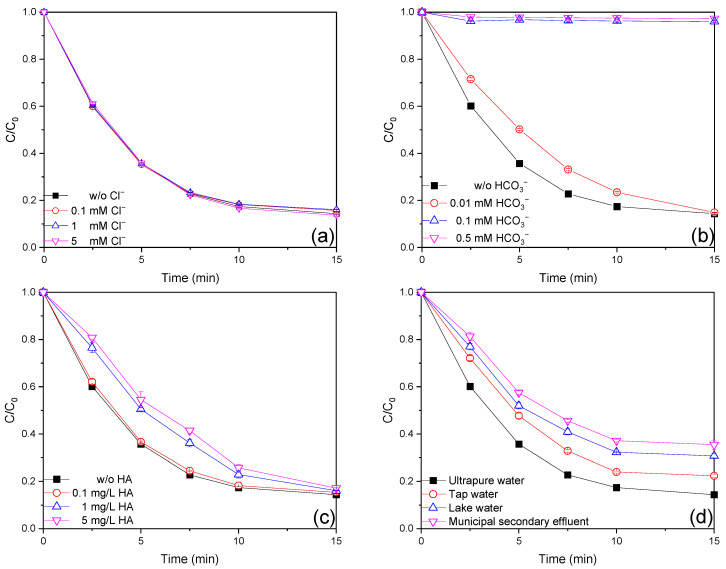
Effect of initial (**a**) Cl^−^, (**b**) HCO_3_^−^, (**c**) HA concentration, and (**d**) real water matrix on AO7 degradation efficiencies in the Fe(III)/MBS process (reaction condition: [AO7]_0_ = 0.02 mM, [Fe(III)]_0_ = 0.01 mM, [MBS]_0_ = 0.1 mM, T = 25 °C).

Chloride (Cl^−^), a common constituent of wastewater, is thought to play a vital role in AOPs as a scavenger or precursor to the reactive species [36]. As shown in Figure 4a, the AO7 removal efficiency with the addition of 0.1, 1, and 5 mM Cl^−^ was 84.3%, 83.9%, and 86.3%, respectively. In addition, the *k_obs_* values remained around 0.180 min^−1^ within 15 min with the tested dosages of Cl^−^ (Figure S9a). It is obvious that the presence of Cl^−^ in the tested concentrations had a negligible effect on the removal of AO7, which could be attributed to Cl^−^ could react with HO^•^ and SO_4_^•−^ (*k*_Cl−,HO•_ = 4.3 × 10^10^ M^−1^ s^−1^, *k*_Cl−,SO4•−_ = 4.7 × 10^8^ M^−1^ s^−1^ [37]) to generate the reactive chlorine species (RCS, including Cl^•^, Cl_2_^•–^ and HOCl^•−^) (Equations (2)–(5)). RCS can also participate in the degradation of AO7 [38].

(2) $${\mathrm{Cl}}^{-}{+\mathrm{SO}}_{4}^{•-}⟶{\mathrm{Cl}}^{•}{+\mathrm{SO}}_{4}^{2-}$$

(3) $${\mathrm{Cl}}^{-}+{\mathrm{Cl}}^{•}⟶{{\mathrm{Cl}}_{2}}^{•-}$$

(4) $${\mathrm{Cl}}^{-}{+\mathrm{HO}}^{•}⟶{\mathrm{HOCl}}^{•-}$$

(5) $${\mathrm{HOCl}}^{•-}+H^{+}⟶{\mathrm{Cl}}^{•}{+H}_{2}O$$

Generally, bicarbonate (HCO_3_^−^) has a negative effect on AOPs as it is a reactive species quencher. As shown in Figure 4b, the AO7 removal efficiency with 0.01, 0.1, and 0.5 mM HCO_3_^−^ was 85.1%, 4.0%, and 2.7%, respectively. In addition, the *k_obs_* values decreased from 0.179 min^−1^ to 0.131 min^−1^, 0.016 min^−1^, and 0.013 min^−1^ when 0.01, 0.1, and 0.5 mM HCO_3_^−^ were added, respectively (Figure S9b). Bicarbonate at low concentration (0.01 mM) had a negligible impact, whereas high concentrations (0.1 mM and 0.5 mM) retarded AO7 removal remarkably in the Fe(III)/MBS process. The observed results may be attributed to the possibility of the addition of HCO_3_^−^ to elevate the pH value, which may adversely affect the removal of AO7. In addition, HCO_3_^−^ could react with HO^•^ and SO_4_^•−^ (*k*_HCO3−,HO•_ = 8.7 × 10^6^ M^−1^ s^−1^, *k*_HCO3−,SO4•−_ = 5.95 × 10^6^ M^−1^ s^−1^ [37]) to form CO_3_^•−^ (Equations (6) and (7)) with lower redox potential (1.59 V) and higher selectivity, making it less available for reacting with AO7 [39].

(6) $${\mathrm{HCO}}_{3}^{-}{+\mathrm{SO}}_{4}^{•-}⟶{\mathrm{CO}}_{3}^{•-}{+\mathrm{HSO}}_{4}^{-}$$

(7) $${\mathrm{HCO}}_{3}^{-}{+\mathrm{HO}}^{•}⟶{\mathrm{CO}}_{3}^{•-}{+H}_{2}O$$

Natural organic matter (NOM) is ubiquitous in actual water at concentrations of 1–10 mg/L [37]. It is well-known that the presence of NOM can reduce the steady-state concentration of reactive species. As shown in Figure 4c, the AO7 removal efficiencies with the addition of 0.1, 1, and 5 mg/L HA were 84.6%, 83.9%, and 82.9%, respectively. Meanwhile, the *k_obs_* values decreased from 0.179 min^−1^ to 0.174 min^−1^, 0.129 min^−1^, and 0.124 min^−1^ in the presence of 0.1, 1, and 5 mg/L HA, respectively (Figure S9c). On the one hand, HA competitively reacted with HO^•^ and SO_4_^•−^ (Equations (8) and (9), *k*_HA,HO•_ = 2.95 × 10^8^ M^−1^ s^−1^, *k*_HA,SO4•−_ = 6.4 × 10^7^ M^−1^ s^−1^ [37]), making it less likely to react with AO7 [40]. Additionally, HA complexed with Fe(III) [37], resulting in it being less available for S(IV) activation.

(8) $${\mathrm{HA}+\mathrm{SO}}_{4}^{•-}⟶{\mathrm{HA}}_{\mathrm{products}}{+\mathrm{HSO}}_{4}^{-}$$

(9) $${\mathrm{HA}+\mathrm{HO}}^{•}⟶{\mathrm{HA}}_{\mathrm{products}}{+H}_{2}O$$

Further experiments were conducted to evaluate the efficacy of the Fe(III)/MBS process in three real water matrices. As shown in Figure 4d, totals of 77.6%, 69.3%, and 64.5% of AO7 removal were achieved in tap water, lake water, and filtered municipal secondary effluent, respectively. Based on the concentration of each component in the three real water matrices (Table S6) and the previous discussions, it was postulated that NOM and Cl^−^ were primarily responsible for the retarded AO7 removal efficiency in the Fe(III)/MBS process. It is reasonable to deduce that waters with low concentrations of NOM and Cl^−^, or those that have undergone effective pre-treatment to remove NOM and Cl^−^, will be best suited to achieving significant removal efficiency when the Fe(III)/MBS process is employed to degrade organic contaminants in actual waters.

### 2.5. Possible Degradation Pathway of AO7

In order to ascertain the alterations in the molecular and structural attributes of AO7 throughout the Fe(III)/MBS process, the UV–visible spectrum of the treated AO7 solution was subjected to observation. As shown in Figure S10a, the four distinctive peaks at 228, 310, 430, and 484 nm represent the benzene ring, naphthalene ring, hydrazone form, and azo form of AO7, respectively. The gradual decrease in their absorbance in both the UV and visible regions (Figure S10b) is indicative of the destruction of the AO7 structure. Figure S10b demonstrates a diminishing absorbance at these four wavelengths, following the sequence 484 nm ≥ 430 nm > 310 nm ≥ 228 nm. This pattern suggests that SO_4_^•−^ and HO^•^ engage in the selective attack of distinct conjugate moieties of AO7, with the azo bond, naphthalene rings, and benzene ring undergoing the reaction in that order [41]. This finding is markedly different from observations made in the photo–Fe(II) sulfite process, which indicates that the rate of degradation of the naphthalene and benzene rings is significantly faster than the rate of decolorization observed in that process [42], indicating that the rate of destruction of the naphthalene and benzene rings was significantly faster than the rate of decolorization observed in the aforementioned photo–Fe(II) sulfite process. Furthermore, the inadequate TOC removal (14.7%, Figure S11) within 60 min supported this conclusion, indicating that the Fe(III)/MBS process was more efficacious in decolorization than in mineralization.

From the HOMO and LUMO orbits of the AO7 molecule in Figure S12, it can be observed that the electrophilic reaction activity of –SO_3_^−^ of AO7 in the HOMO orbital was higher than in the other region of the molecule. Conversely, the nucleophilic reactivity of the azo double bond, N–C bond, and naphthalene ring in the LUMO orbital were significantly higher. The findings indicate that the AO7 molecule contains a number of reactive components that are susceptible to external reactive species [43]. FED calculations were conducted to identify potential sites for AO7 degradation, where a larger 2FED^2^_HOMO_ is associated with a high tendency to extract electrons, and reactive species are more likely to attack atoms with a higher value of FED^2^_HOMO_ + FED^2^_LUMO_ (Table S7). The results of the HOMO and LUMO orbit images and FED calculations of AO7 demonstrate that the most reactive sites were located at N1, N2, C18, C20, and C27.

**Figure 5:**
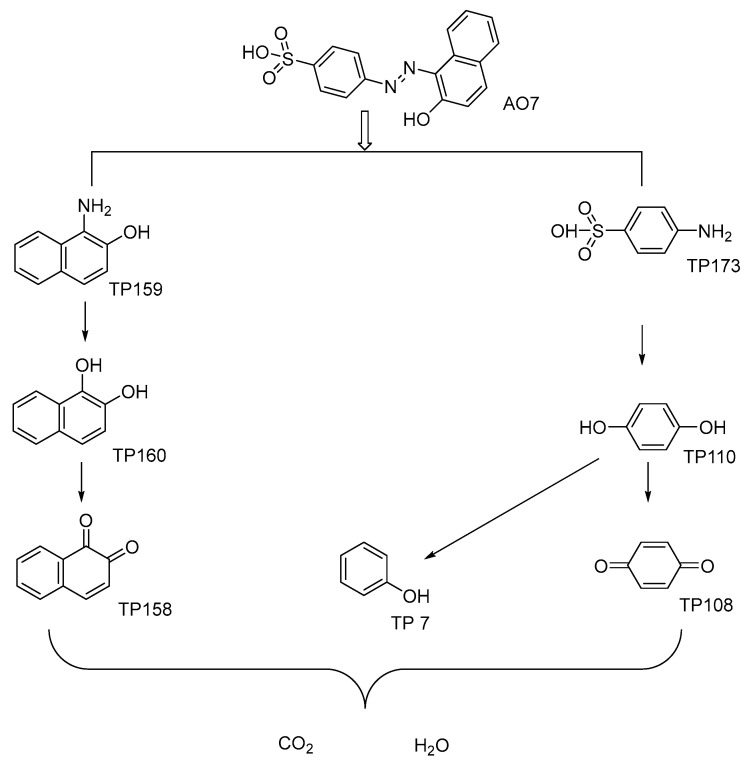
Possible degradation pathway of AO7 in the Fe(III)/MBS process.

To elucidate the mechanism underlying AO7 decolorization in the Fe(III)/MBS process, transformation products resulting from AO7 removal were identified using gas chromatography–mass spectrometry (GC–MS) analysis. This analysis confirmed the presence of seven major transformation products. The identified transformation products (Table S8 and Figure S13) are predominantly aliphatic organics with distinctive oxygen groups, which may have been formed by the reaction of reactive radicals with the naphthalene and benzene rings of the AO7 molecules [44]. In general, SO_4_^•−^ reacts with organic compounds primarily via selective electron transfer, whereas HO^•^ predominantly reacts through electrophilic addition and hydrogen abstraction. The attack of AO7 by HO^•^ initially resulted in the destruction of the N=N bond, leading to the formation of 1-amino-2-naphthol (TP159) and 4-aminobenzenesulfanilic acid (TP173). Subsequently, the reaction of 1-amino-2-naphthol with SO_4_^•−^ and HO^•^ resulted in the formation of 1,2-naphthalenediol (TP160) and 1,2-naphthalenedione (TP158). Furthermore, 4-aminobenzenesulfonic acid was transformed into 1,4-benzenediol (TP110), phenol (TP94), and 1,4-benzoquinone (TP108) by SO_4_^•−^ and HO^•^ through a series of electron transfer, hydroxylation, and hydrogen abstraction reactions [45]. In light of the above results, potential pathways for AO7 degradation in the Fe(III)/MBS process are proposed in Figure 5, including N=N cleavage, hydroxylation, and hydrogen abstraction.

## 3. Materials and Methods

### 3.1. Materials

All chemical reagents were of at least analytical grade and were utilized in their original state. AO7, rhodamine B, methyl orange, and tetracycline were supplied by Aladdin (Shanghai, China). The PMS, phenol, acetaminophen, and sulfamethoxazole were obtained from Sigma-Aldrich (St. Louis, MO, USA). The MBS, sodium sulfite, sodium bisulfite, PDS, hydrogen peroxide, ferric chloride, ferrous sulfate, sulfuric acid, sodium hydroxide, ammonium ferrous sulfate, 1,10-phenanthroline, acetic acid, ammonium acetate, disodium hydrogen phosphate, sodium chloride, sodium bicarbonate, and humic acid were purchased from Sinopharm (Shanghai, China). The methanol, ethanol (EtOH), *tert*–butyl alcohol (TBA), methyl phenyl sulfoxide (PMSO), methyl phenyl sulfone (PMSO_2_), 5,5–dithiobis (2–nitrobenzoic acid) (DTNB), and formic acid were supplied by J&K Scientific (Beijing, China). The tap water, lake water, and municipal secondary effluent were taken from the laboratory and Caiyue Lake at Nanjing Normal University Campus and a wastewater treatment plant in Chongqing, respectively. Following filtration through glass fiber membranes, the water samples were stored at 4 °C. All solutions were prepared in advance of the experiment using ultrapure water with a resistance of 18.2 MΩ·cm from the Pall Cascada I process (Pall Corporation, Port Washington, NY, USA).

### 3.2. Experimental Procedure

The stock solution of organic contaminants was freshly dissolved in ultrapure water, and the experimental concentration (*C*_0_) of organic contaminants was maintained at 0.02 mM. This concentration is several orders of magnitude higher than those typically found in wastewater samples (ng-μg/L). However, the use of such an initial concentration allows for the evaluation of degradation efficiency within a measurable time scale as well as the detection of reaction products with the analytical techniques employed in this work. Degradation experiments were conducted in a 250 mL glass flask at a controlled temperature of room temperature, with the exception of those involving the reaction temperature effects, which were performed within a constant-temperature water bath shaker unit (THZ–82, Changzhou, China). The experiments were initiated by combining the requisite quantities of organic contaminants, anions or radical scavengers, if applicable, ferric chloride, and MBS in the specified sequence. During the course of the treatment, a rapid shaking motion was employed to ensure complete mixing of the solution. The initial pH of the mixture solution was not modified, except in instances where the effect of pH was deemed pertinent to the study. At the specified reaction time intervals, the appropriate amount of sample was withdrawn and the organic contaminants content was determined as soon as possible.

### 3.3. Analytical Methods

The contents of AO7, rhodamine B, methyl orange, and tetracycline were measured using a UV–Vis spectrophotometer (UV–5500, Metash, Shanghai, China) at the characteristic λ_max_ wavelengths of 484 nm, 554 nm, 464 nm, and 357 nm, respectively. The UV–Vis spectra of treated AO7 samples were recorded by the same equipment between 200 and 600 nm. The concentrations of other organic contaminants (phenol, acetaminophen, sulfamethoxazole, PMSO, and PMSO_2_) were determined using an ultra-high performance liquid chromatography (Agilent 1290, Agilent, Santa Clara, CA, USA) equipped with a UV detector. Separation was performed on an Athena UHPLC C18 column (2.1 × 50 mm, 1.8 μm, CNW, Shanghai, China) using a mobile phase comprising a binary mixture of 0.1% (*v*/*v*) formic acid and methanol at a flow rate of 1.0 mL min^−1^ (Table S3).

The content of MBS was monitored by the DTNB method [30]. The concentration of ferrous ion was determined by the 1,10-phenanthroline method [17]. Gas chromatography–mass spectrometry (GC–MS-QP2020, Shimadzu, Kyoto, Japan) was used for analysis of the generated AO7 intermediates in the Fe(III)/MBS process. The treated AO7 solutions collected at 2.5, 5, 7.5, 10, and 15 min were mixed and then extracted using dichloromethane (5 times, 10 mL each time). The extraction liquids were concentrated to approximately 1.0 mL on a rotary evaporator at 40 °C in a nitrogen atmosphere. The concentrated samples were then transferred to vials and stored in a refrigerator at 4 °C before GC–MS analysis. A DB–5MS capillary column with an inner diameter of 0.25 mm and a length of 30 m was adopted in the separation process. The GC column was operated in a temperature-programmed mode by retaining the temperature at 40 °C for 2 min, ramped first to 100 °C at a 15 °C min^−1^ rate, then raised to 200 °C with a 5 °C min^−1^ rate, and finally programmed to 280 °C at a 20 °C min^−1^ rate, and held at 280 °C for 10 min. Electron impact (EI) mode at 70 eV was used and spectra were obtained in a scan range of 50–500 *m*/*z*. Dissolved oxygen (DO) was determined by a DO meter (JPB–607A, INESA, Shanghai, China). TOC analysis of samples was performed on an Analytik Jena multi N/C 2100 (Analytik Jena, Jena, Germany). Cl^−^, NO_3_^−^, and SO_4_^2−^ were measured with an ion chromatography system (ICS1100, Dionex, Sunnyvale, CA, USA). The mobile phase was 20 mM KOH at a flow rate of 1.0 mL min^−1^. A basic PB–10 pH meter (Sartorius Scientific Instruments, Beijing, China) was used to monitor the pH values. The Gaussian 09 program 6–311++G(2d,2p) was employed to calculate the frontier electron densities (FEDs) of the highest occupied molecular orbital (HOMO) and the lowest unoccupied molecular orbital (LUMO) of AO7 molecules.

## 4. Conclusions

The present study demonstrates that MBS, a low-cost, low-risk, and eco-friendly agent, effectively enhances the removal of AO7 with dissolved Fe(III) at environmentally relevant concentrations (i.e., <1 mg/L). Specifically, under conditions of 0.01 mM Fe(III) and 0.1 mM MBS at 25 °C, the Fe(III)/MBS process achieved a removal efficiency of 85.6% for 0.02 mM AO7. AO7 removal efficiency increased first and then retarded with the elevated Fe(III) or MBS dosage. Additionally, AO7 removal efficiency increased and then decreased with the elevated pH values. Furthermore, HO^•^ and SO_4_^•−^ were the dominant reactive species and their contributions to AO7 degradation were estimated to be 51.59% and 46.45%, respectively. Furthermore, the impact of Cl^−^ was found to be insignificant, whereas the degradation of AO7 was found to be inhibited in the presence of HCO_3_^−^ and humic acid. Satisfactory results were obtained for the removal of AO7 in three real waters. Finally, N=N cleavage, hydroxylation, and hydrogen abstraction were proposed as the AO7 degradation pathways. Overall, our findings contribute valuable insights into the mechanisms of organic contaminant removal in wastewater treatment processes and highlight the potential application of MBS in enhancing the efficacy of such treatments. Further research should assess the optimal conditions for practical application, taking into account the toxicity of MBS and its impact on environment.

## Data Availability

Data is contained within the article and Supplementary Materials.

## Supplementary Materials

The following supporting information can be downloaded at: https://www.mdpi.com/article/10.3390/ijms26030953/s1.

## References

[1] Anipsitakis G.P., Dionysiou D.D. (2003). Degradation of organic contaminants in water with sulfate radicals generated by the conjunction of peroxymonosulfate with cobalt. Environ. Sci. Technol..

[2] Sun S., Pang S., Jiang J., Ma J., Huang Z., Zhang J., Liu Y., Xu C., Liu Q., Yuan Y. (2018). The combination of ferrate(VI) and sulfite as a novel advanced oxidation process for enhanced degradation of organic contaminants. Chem. Eng. J..

[3] Zhou X., Li X., Xiang Y., Zhang H., He C., Xiong Z., Li W., Zhou P., Zhou H., Liu Y. (2024). The application of low-valent sulfur oxy-acid salts in advanced oxidation and reduction processes: A review. Chin. Chem. Lett..

[4] Ren Y., Chu Y., Li N., Lai B., Zhang W., Liu C., Li J. (2023). A critical review of environmental remediation via iron-mediated sulfite advanced oxidation processes. Chem. Eng. J..

[5] Gao Y., Fan W., Zhang Z., Zhou Y., Zeng Z., Yan K., Ma J., Hanna K. (2022). Transformation mechanisms of iopamidol by iron/sulfite systems: Involvement of multiple reactive species and efficiency in real water. J. Hazard. Mater..

[6] Zhou D., Chen L., Li J., Wu F. (2018). Transition metal catalyzed sulfite auto-oxidation systems for oxidative decontamination in waters: A state-of-the-art minireview. Chem. Eng. J..

[7] Farinelli G., Gil A.G., Marugan J., Minella M., Fabbri D., Laurenti E., Tiraferri A., Vione D. (2024). The dominant role of the peroxymonosulfate radical for removing contaminants in a Fenton process with metabisulfite. Environ. Chem. Lett..

[8] Hayon E., Treinin A., Wilf J. (1972). Electronic spectra, photochemistry, and autoxidation mechanism of the sulfite-bisulfite-pyrosulfite systems. SO_2_^−^, SO_3_^−^, SO_4_^−^, and SO_5_^−^ radicals. J. Am. Chem. Soc..

[9] Ahmadi F., Lee Y.H., Lee W.H., Oh Y.K., Park K.K., Kwak W.S. (2018). Preservation of fruit and vegetable discards with sodium metabisulfite. J. Environ. Manag..

[10] Gromboni C.F., Donati G.L., Matos W.O., Neves E.F.A., Nogueira A.R.A., Nóbrega J.A. (2010). Evaluation of metabisulfite and a commercial steel wool for removing chromium(VI) from wastewater. Environ. Chem. Lett..

[11] Ma J., Liu C., Chen K. (2020). Removal of Cr(VI) species from water with a newly-designed adsorptive treatment train. Sep. Purif. Technol..

[12] Liu T., Liu Y., Zhang H., Zhou P., Li W., Xiong Z., He C., Du Y., Yao G., Lai B. (2025). New insights on the efficiency and mechanism of dissolved oxygen regulating the redox removal of organic pollutants by S(IV) system. Sep. Purif. Technol..

[13] Liu T., Liu Y., Zhou P., Xiong Z., Zhang H., He C., Du Y., Yao G., Lai B. (2022). New insight of zero-valent iron activation S(IV) in absence of external hydrogenation ions for decomposing micropollutants: Two radical generation pathways depending on O_2_. J. Hazard. Mater..

[14] Savia F., Adesina A.O., Carena L., Vione D. (2023). Assessment of Fenton systems based on metabisulphite as a low-cost alternative to hydrogen peroxide. J. Environ. Chem. Eng..

[15] Wilfert P., Kumar P.S., Korving L., Witkamp G.-J., van Loosdrecht M.C.M. (2015). The Relevance of Phosphorus and Iron Chemistry to the Recovery of Phosphorus from Wastewater: A Review. Environ. Sci. Technol..

[16] Zhang Y., Peng G., Yan Y., Meng X., Gong W. (2025). Highly Efficient Removal of Organic Pollutants with HCO_3_^−^-Enhanced Ru(III)/NaClO Process. Int. J. Mol. Sci..

[17] Qi C., Wen Y., Zhao Y., Dai Y., Li Y., Xu C., Yang S., He H. (2022). Enhanced degradation of organic contaminants by Fe(III)/peroxymonosulfate process with l-cysteine. Chin. Chem. Lett..

[18] Yuan Y., Yang S., Zhou D., Wu F. (2016). A simple Cr(VI)–S(IV)–O_2_ system for rapid and simultaneous reduction of Cr(VI) and oxidative degradation of organic pollutants. J. Hazard. Mater..

[19] Liu T., Xie Z., Zhou P., Xiong Z., Zhang H., Pan Z., Liu Y., Lai B. (2022). Enhanced degradation of carbamazepine by iron/S(IV) system using a novel S(IV) source. Chem. Eng. J..

[20] Wang Z., Bai F., Cao L., Yue S., Wang J., Wang S., Ma J., Xie P. (2022). Activation of sulfite by ferric ion for the degradation of 2,4,6-tribromophenol with the addition of sulfite in batches. Chin. Chem. Lett..

[21] Dong H., Wei G., Yin D., Guan X. (2020). Mechanistic insight into the generation of reactive oxygen species in sulfite activation with Fe(III) for contaminants degradation. J. Hazard. Mater..

[22] Zhou D., Yuan Y., Yang S., Gao H., Chen L. (2015). Roles of oxysulfur radicals in the oxidation of acid orange 7 in the Fe(III)–sulfite system. J. Sulfur Chem..

[23] Yuan Y., Luo T., Xu J., Li J., Wu F., Brigante M., Mailhot G. (2019). Enhanced oxidation of aniline using Fe(III)-S(IV) system: Role of different oxysulfur radicals. Chem. Eng. J..

[24] Yu Y., Li S., Peng X., Yang S., Zhu Y., Chen L., Wu F., Mailhot G. (2016). Efficient oxidation of bisphenol A with oxysulfur radicals generated by iron-catalyzed autoxidation of sulfite at circumneutral pH under UV irradiation. Environ. Chem. Lett..

[25] Wang S., Wang G., Fu Y., Wang H., Liu Y. (2020). A simple Fe^3+^/bisulfite system for rapid degradation of sulfamethoxazole. RSC Adv..

[26] Xie P., Zhang L., Wang J., Zou Y., Wang S., Yue S., Wang Z., Ma J. (2020). Transformation of tetrabromobisphenol a in the iron ions-catalyzed auto-oxidation of HSO_3_^2−^/SO_3_^2−^ process. Separ. Purif. Technol..

[27] Wang C., Huo Y., Lu W., Shen X., Xu L. (2024). A comparative study of sulfite activation using different transition metal ions for the degradation of bisphenol A. J. Environ. Chem. Eng..

[28] Zeng H., Cheng Y., Repo E., Yu X., Xing X., Zhang T., Zhao X. (2022). Trace Iron as single-electron shuttle for interdependent activation of peroxydisulfate and HSO_3_^−^/O_2_ enables accelerated generation of radicals. Water Res..

[29] Kuo D.T.F., Kirk D.W., Jia C.Q. (2006). The chemistry of aqueous S(IV)-Fe-O_2_ system: State of the art. J. Sulfur Chem..

[30] Qi C., Liu X., Li Y., Lin C., Ma J., Li X., Zhang H. (2017). Enhanced degradation of organic contaminants in water by peroxydisulfate coupled with bisulfite. J. Hazard. Mater..

[31] Chen L., Peng X., Liu J., Li J., Wu F. (2012). Decolorization of Orange II in Aqueous Solution by an Fe(II)/sulfite System: Replacement of Persulfate. Ind. Eng. Chem. Res..

[32] Ding W., Huang X., Zhang W., Wu F., Li J. (2019). Sulfite activation by a low-leaching silica-supported copper catalyst for oxidation of As(III) in water at circumneutral pH. Chem. Eng. J..

[33] Wang H., Wang S., Liu Y., Fu Y., Wu P., Zhou G. (2019). Degradation of diclofenac by Fe(II)-activated bisulfite: Kinetics, mechanism and transformation products. Chemosphere.

[34] Fang L., Chen H., Liao P., Ma Y., Lv Y. (2024). Enhanced degradation of tetracycline hydrochloride by microwave-activated sulfites: Influencing factors and mechanisms. J. Water Process Eng..

[35] Gong W., He D., Wang X., Yan Y., Dionysiou D.D., Blaney L., Peng G. (2024). The role of Fe(IV) in the zero-valent iron biochar activated persulfate system for treatment of contaminants of emerging concern. Chem. Eng. J..

[36] Xue Y., Wang Z., Naidu R., Bush R., Yang F., Liu J., Huang M. (2022). Role of halide ions on organic pollutants degradation by peroxygens-based advanced oxidation processes: A critical review. Chem. Eng. J..

[37] Yang X., Rosario-Ortiz F.L., Lei Y., Pan Y., Lei X., Westerhoff P. (2022). Multiple Roles of Dissolved Organic Matter in Advanced Oxidation Processes. Environ. Sci. Technol..

[38] Ali J., Shahzad A., Wang J., Ifthikar J., Lei W., Aregay G.G., Chen Z., Chen Z. (2021). Modulating the redox cycles of homogenous Fe(III)/PMS system through constructing electron rich thiomolybdate centres in confined layered double hydroxides. Chem. Eng. J..

[39] Wang J., Wang S. (2021). Effect of inorganic anions on the performance of advanced oxidation processes for degradation of organic contaminants. Chem. Eng. J..

[40] Qi C., Yu G., Huang J., Wang B., Wang Y., Deng S. (2018). Activation of persulfate by modified drinking water treatment residuals for sulfamethoxazole degradation. Chem. Eng. J..

[41] Chen Y., Tong Y., Liu Z., Huang L.-Z., Yuan J., Xue Y., Fang Z. (2019). Enhanced degradation of Orange II using a novel UV/persulfate/sulfite system. Environ. Chem. Lett..

[42] Zhou D., Chen L., Zhang C., Yu Y., Zhang L., Wu F. (2014). A novel photochemical system of ferrous sulfite complex: Kinetics and mechanisms of rapid decolorization of Acid Orange 7 in aqueous solutions. Water Res..

[43] Huo Y., Zheng H., Jiang Y., Chen H., Cao W., Mameda N., Nghiem L.D., Zhang X., Liu Q. (2023). Comparison and Characterization of Nitrogen/Sulfur-Doped Activated Carbon for Activating Peroxydisulfate to Degrade Acid Orange 7: An Experimental and Theoretical Study. Ind. Eng. Chem. Res..

[44] Liu Y.-C., Liu X., Wang X., Li Z.-H., Chen C.-L., Xiang Z. (2023). Hybrid persulfate/sonocatalysis for degradation of acid orange 7 in the presence of Ag_2_O/CuWO_4_ composite: Operating parameters and sonocatalytic mechanism. J. Clean. Prod..

[45] Cai C., Zhang H., Zhong X., Hou L. (2014). Electrochemical enhanced heterogeneous activation of peroxydisulfate by Fe-Co/SBA-15 catalyst for the degradation of Orange II in water. Water Res..

