# Supplementary material for: Activation of Metabisulfite by Dissolved Fe(III) at Environmentally Relevant Concentrations for Organic Contaminants Degradation
Source: Int J Mol Sci. 2025 Jan 23;26(3):953. doi: 10.3390/ijms26030953 (PMC11817393; doi:10.3390/ijms26030953)
Supplement: Supplementary file 1 [file ijms-26-00953-s001.zip › ijms-3411693-supplementary.pdf]

Supporting information

Activation of Metabisulfite by Dissolved Fe(III) at Environmentally Relevant Concentrations for Organic Contaminants Degradation

Jianan Chen [1,†], Longjiong Chen [1,†], Leliang Wu [1], Chengyu Yan [1], Ningxin Sun [1], Guilong Peng [2,*], Shaogui Yang [1], Huan He [1] and Chengdu Qi [1,3,*]

[1] School of Environment, Jiangsu Province Engineering Research Center of Environmental Risk Prevention and Emergency Response Technology, Jiangsu Engineering Lab of Water and Soil Eco–remediation, Nanjing Normal University, Nanjing 210023, China
[2] State Key Laboratory of Resource Insects, College of Sericulture, Textile and Biomass Sciences, Southwest University, Chongqing 400715, China
[3] Hubei Key Laboratory of Mineral Resources Processing and Environment, Wuhan University of Technology, Wuhan 430070, China
* Correspondence: pengguilong@swu.edu.cn (G.P.); qichengdu@njnu.edu.cn (C.Q.)
† These authors contributed equally to this work.

Table S1. Main reactions involved in the Fe(III)/MBS process.

No.	Reactions	Rate/equilibrium constants (k/$M^{-1}s^{-1}$) [a]
1	$S_2O_5^{2-} + H_2O \rightleftharpoons 2HSO_3^-$	
2	$Fe^{3+} + HSO_3^- \rightarrow FeSO_3^+ + H^+$	log k= 2.45
3	$FeSO_3^+ \rightarrow Fe^{2+} + SO_3^{\bullet-}$	0.19 s^{-1}
4	$Fe^{2+} + HSO_3^- \rightarrow FeHSO_3^+$	10^4 M^{-1}
5	$4FeHSO_3^+ + O_2 \rightarrow 4FeSO_3^+ + 2H_2O$	1.69×10^3
6	$SO_3^{\bullet-} + O_2 \rightarrow SO_5^{\bullet-}$	1.5×10^9
7	$SO_5^{\bullet-} + HSO_3^- \rightarrow SO_3^{\bullet-} + HSO_5^-$	$\leq3\times10^5$
8	$SO_5^{\bullet-} + HSO_3^- \rightarrow SO_4^{\bullet-} + HSO_4^-$	1.2×10^4
9	$SO_4^{\bullet-} + HSO_3^- \rightarrow SO_3^{\bullet-} + HSO_4^-$	7.5×10^8
10	$SO_4^{\bullet-} + H_2O \rightarrow HO^\bullet + HSO_4^-$	1.1×10^1
11	$SO_4^{\bullet-} + OH^- \rightarrow HO^\bullet + SO_4^{2-}$	1.4×10^7
12	$EtOH + HO^\bullet \rightarrow$ product	$(1.6–2.2)\times10^9$
13	$EtOH + SO_4^{\bullet-} \rightarrow$ product	$(1.6–7.7)\times10^7$
14	$EtOH + SO_5^{\bullet-} \rightarrow$ product	$\leq10^3$
15	$TBA + HO^\bullet \rightarrow$ product	$(4.2–7.6)\times10^8$
16	$TBA + SO_4^{\bullet-} \rightarrow$ product	$(4.0–9.1)\times10^5$
17	$TBA + SO_5^{\bullet-} \rightarrow$ product	$\leq10^3$
18	$AO7 + HO^\bullet \rightarrow$ product	1.2×10^{10}
19	$AO7 + SO_4^{\bullet-} \rightarrow$ product	8.07×10^9
20	$AO7 + SO_5^{\bullet-} \rightarrow$ product	2.1×10^6

[a] Second order rate constants are given unless otherwise indicated.

Table S2. Studies published related to the Fe(III) catalyzed S(IV) auto-oxidation of organic contaminants (OCs).

OCs	Fe(III)	S(IV)	T (°C)	pH	Removal efficiency	k_{obs} (min^{-1})	reference
0.029 mM acid orange 7	0.1 mM	1 mM SO_3^{2-}	25±2	3.0	54.7% in 20 min	0.151	[1]
0.004 mM bisphenol A	0.1 mM	1 mM SO_3^{2-}		6.0	70.6% in 60 min	0.212	[2]
0.01 mM aniline	0.1 mM	1 mM SO_3^{2-}	25	4.0	65% in 30 min	0.132	[3]
0.005 mM carbamazepine	0.1 mM	0.5 mM SO_3^{2-}	20±1	3.0	75.6% in 10 min	0.372	[4]
0.001 mM sulfamethoxazole	0.01 mM	0.1 mM HSO_3^-	25	4.0	100% in 20 min	0.850	[5]
0.01 mM tetrabromobisphenol a	0.04 mM	0.4 mM SO_3^{2-}	25	4.0	72.0% in 20 min	0.191	[6]
0.005 mM iopamidol	0.05 mM	0.3 mM SO_3^{2-}	25±1	4.0	85.0% in 4 min	1.01	[7]
0.008 mM carbamazepine	0.06 mM	0.1 mM $S_2O_5^{2-}$	25	3.5	86.2% in 20 min	0.512	[8]
0.01 mM 2,4,6-tribromophenol	0.1 mM	0.4 mM SO_3^{2-}	25	4.0	70.2% in 6 min	0.473	[9]
0.01 mM bisphenol A	0.1 mM	1 mM SO_3^{2-}	23±1	3.5	53.0% in 15 min	0.072	[10]
0.02 mM acid orange 7	0.01 mM	0.1 mM $S_2O_5^{2-}$	25	4.5	85.6% in 15 min	0.179	This study

Table S3. Details of analytical methods of UPLC for organic contaminants.

Organic contaminants	The volume ratio of mobile phase	Detection wavelength (nm)
phenol	methanol : 0.1% formic acid = 30 : 70	220
acetaminophen	methanol : 0.1% formic acid = 25 : 75	243
sulfamethoxazole	methanol : 0.1% formic acid = 30 : 70	264
methyl phenyl sulfoxide	methanol : 0.1% formic acid = 15 : 85	230
methyl phenyl sulfone	methanol : 0.1% formic acid = 15 : 85	215

Table S4. Effect of EtOH and TBA concentration on the AO7 degradation rate constants (k_{AO7}) and the derived contributions of radicals in the Fe(III)/MBS process.

EtOH/TBA concentrations (mM)	k_{AO7} (min^{-1})		The contributions of radicals (%)			
	EtOH	TBA	$SO_4^{\bullet-}$+$HO^{\bullet}$	$HO^{\bullet}$	$SO_4^{\bullet-}$	other
0	0.179	0.179	-	-	-	-
1	0.109	0.164	38.95	8.08	30.87	61.05
10	0.042	0.145	76.43	18.85	57.58	23.57
100	0.013	0.087	92.59	51.59	41.00	7.41
500	0.003	0.056	98.04	68.57	29.47	1.96

Table S5. Values of c*k* between scavengers/AO7 and radicals.

EtOH/TBA concentrations (mM)	ck (s^{-1})								
	$c_{EtOH}k_{HO•}$	$c_{EtOH}k_{SO4•-}$	$c_{EtOH}k_{SO5•-}$	$c_{TBA}k_{HO•}$	$c_{TBA}k_{SO4•-}$	$c_{TBA}k_{SO5•-}$	$c_{AO7}k_{HO•}$	$c_{AO7}k_{SO4•-}$	$c_{AO7}k_{SO5•-}$
0	0	0	0	0	0	0			
1	$(1.6–2.2)\times10^{6}$	$(1.6–7.7)\times10^{4}$	≤1	$(4.2–7.6)\times10^{5}$	$(4.0–9.1)\times10^{2}$	≤1			
10	$(1.6–2.2)\times10^{7}$	$(1.6–7.7)\times10^{5}$	≤10	$(4.2–7.6)\times10^{6}$	$(4.0–9.1)\times10^{3}$	≤10	2.4×10^{5}	1.61×10^{5}	42
100	$(1.6–2.2)\times10^{8}$	$(1.6–7.7)\times10^{6}$	≤100	$(4.2–7.6)\times10^{7}$	$(4.0–9.1)\times10^{4}$	≤100			
500	$(0.8–1.1)\times10^{9}$	$(0.8–1.5)\times10^{7}$	≤500	$(2.1–3.8)\times10^{8}$	$(2.0–4.5)\times10^{5}$	≤500			

Table S6. Physico-chemical parameters of real water samples.

	pH	UV254	Cl^- (mg/L)	NO_3^- (mg/L)	SO_4^{2-} (mg/L)	TOC (mg/L)
Ultrapure water	5.50	0	n.d.	n.d.	n.d.	n.d.
Tap water	8.01	0.012	25.4	5.9	39.6	2.39
Lake water	7.92	0.068	12.2	<0.1	22.0	16.68
Municipal secondary effluent	7.68	0.061	93.0	46.7	82.6	10.11

n.d.: Not detected.

Table S7. The FED^2_{HOMO}, FED^2_{LUMO}, $2FED^2_{HOMO}$ and FED^2_{HOMO}+FED^2_{LUMO} values of AO7 molecule.

	FED^2_{HOMO}	FED^2_{LUMO}	$2FED^2_{HOMO}$	FED^2_{HOMO}+FED^2_{LUMO}
N1	0.074	0.189	0.147	**0.263**
N2	0.096	0.189	0.192	**0.285**
C3	0.030	0.048	0.060	0.078
C4	0.028	0.045	0.055	0.073
C5	0.023	0.049	0.046	0.072
C6	0.008	0.024	0.016	0.031
H7	0.002	0.005	0.005	0.007
C8	0.009	0.015	0.019	0.025
H9	0.004	0.008	0.007	0.011
C10	0.034	0.059	0.067	0.093
H11	0.000	0.002	0.001	0.003
H12	0.001	0.001	0.001	0.002
S13	0.004	0.010	0.007	0.013
O14	0.007	0.006	0.015	0.013
O15	0.001	0.002	0.003	0.003
O16	0.004	0.005	0.008	0.009
Na17	0.000	0.001	0.000	0.001
C18	0.135	0.038	0.270	**0.173**
C19	0.021	0.027	0.043	0.049
C20	0.089	0.068	0.177	**0.157**
C21	0.075	0.009	0.149	0.083
C22	0.020	0.014	0.041	0.034
C23	0.022	0.027	0.044	0.049
C24	0.029	0.017	0.059	0.046
H25	0.008	0.003	0.016	0.011
C26	0.061	0.012	0.121	0.073
C27	0.076	0.078	0.151	**0.154**
H28	0.001	0.002	0.003	0.003
C29	0.056	0.004	0.112	0.060
H30	0.002	0.002	0.005	0.004
H31	0.005	0.001	0.010	0.006
H32	0.007	0.009	0.013	0.016
H33	0.005	0.000	0.010	0.005
O34	0.058	0.028	0.116	0.087
H35	0.005	0.002	0.010	0.007

Bold indicates that the site is more vulnerable to being attacked by reactive species.

Table S8. AO7 and its transformation products in the Fe(III)/MBS process identified by GC-MS.

Products	m/z	Chemical name	Chemical structure
AO7	328.1	acid orange 7	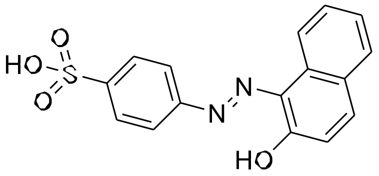
TP159	159.1	1-amino-2-naphthhol	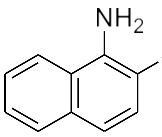
TP173	173.0	4-aminobenzenesulfanilic acid	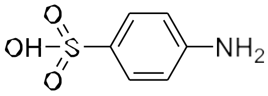
TP110	110.0	1,4-benzenediol	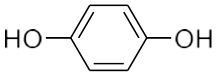
TP108	108.0	1,4-benzoquinone	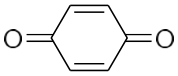
TP160	160.1	1,2-naphthalenediol	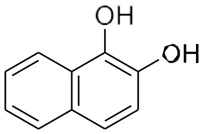
TP158	158.0	1,2-naphthalenedione	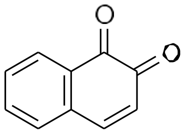
TP94	94.0	phenol	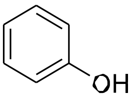

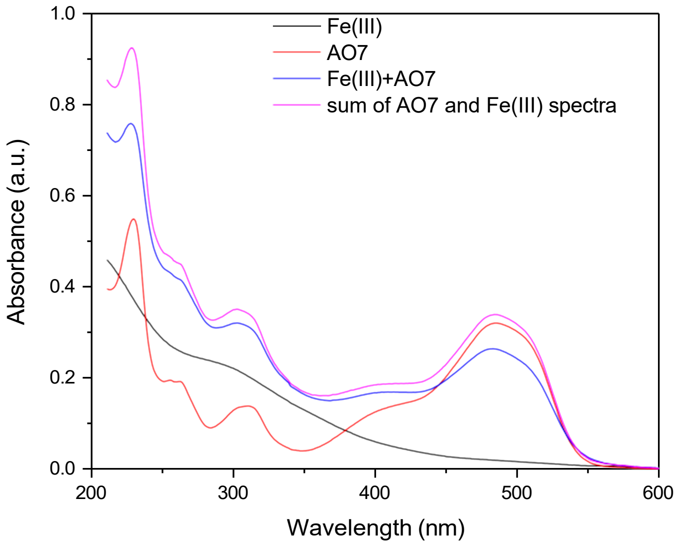

Figure S1. UV-Vis absorption spectra of AO7 (0.02 mM), Fe(III) (0.1 mM), and the mixture of AO7 and Fe(III) in water. The arithmetic sum of the AO7 and Fe(III) spectrum is compared with the spectrum of their mixture (AO7+Fe(III)).

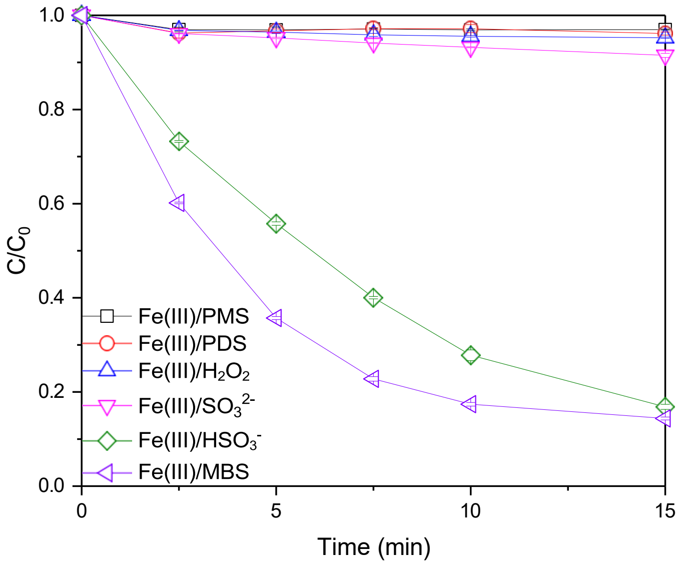

Figure S2. Degradation efficiency of AO7 in the Fe(III) activate oxidant processes (Reaction condition: $[AO7]_0 = 0.02$ mM, $[Fe(III)]_0 = 0.01$ mM, $[PDS]_0 = [H_2O_2]_0 = [MBS]_0 = 0.1$ mM, $[PMS]_0 = [SO_3^{2-}]_0 = [HSO_3^-]_0 = 0.2$ mM, T = 25 °C).

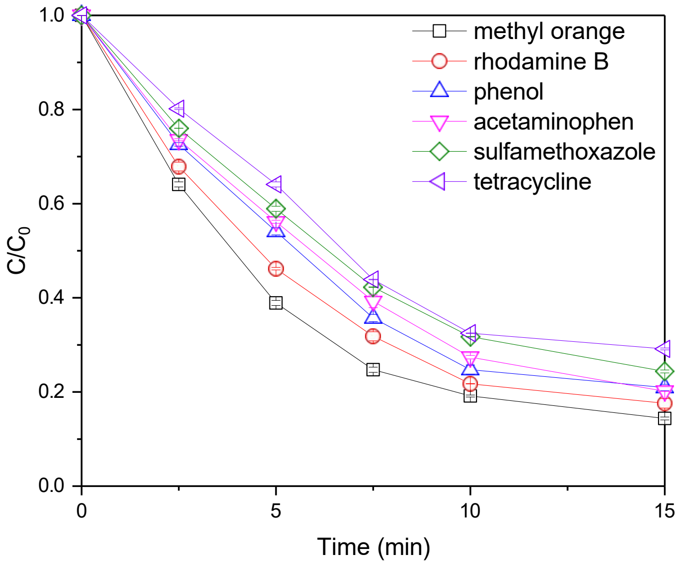

Figure S3. Degradation efficiency of other organic contaminants in the Fe(III)/MBS process (Reaction condition: [organic contaminants]$_0$ = 0.02 mM, [Fe(III)]$_0$ = 0.01 mM, [MBS]$_0$ = 0.1 mM, T = 25 °C).

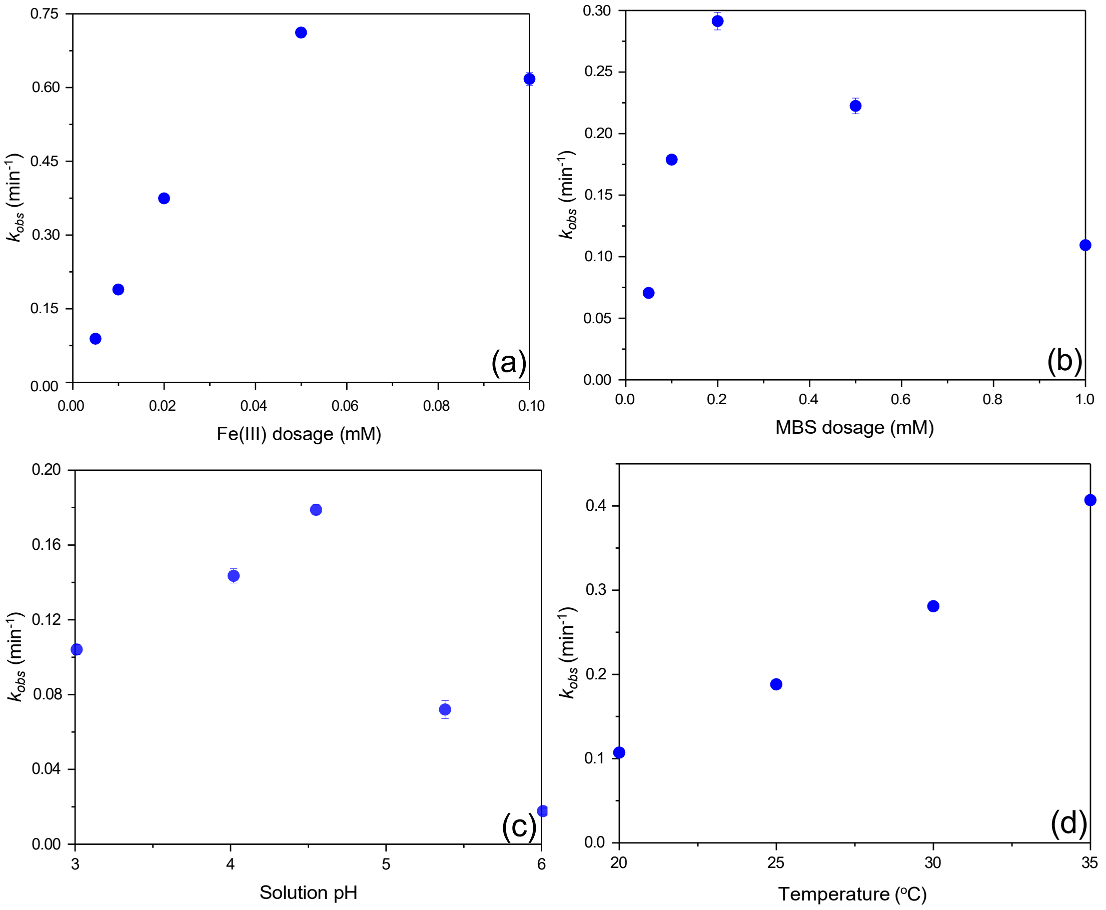

Figure S4. Effect of initial (a) Fe(III), (b) MBS concentration, (c) solution pH, and (d) reaction temperature on AO7 degradation rate constants (k_{obs}) in the Fe(III)/MBS process (Except for the investigated parameter, the other parameters were fixed at: $[AO7]_0 = 0.02$ mM, $[Fe(III)]_0 = 0.01$ mM, $[MBS]_0 = 0.1$ mM, T = 25 °C).

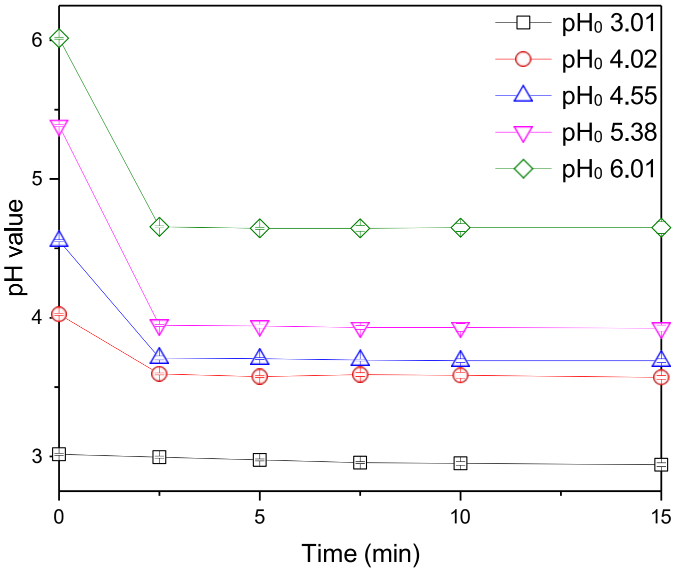

Figure S5. Evolution of pH values during AO7 degradation under the different initial solution pH in the Fe(III)/MBS process (Reaction condition: $[AO7]_0 = 0.02$ mM, $[Fe(III)]_0 = 0.01$ mM, $[MBS]_0 = 0.1$ mM, T = 25 °C).

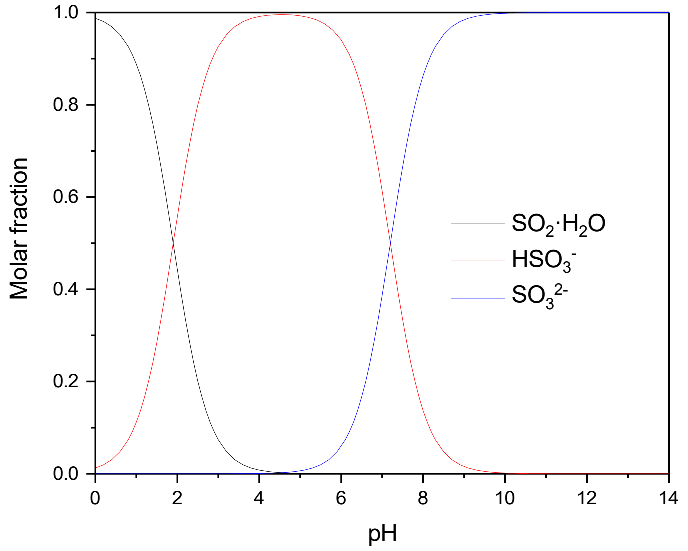

Figure S6. Distribution species of MBS at different pH.

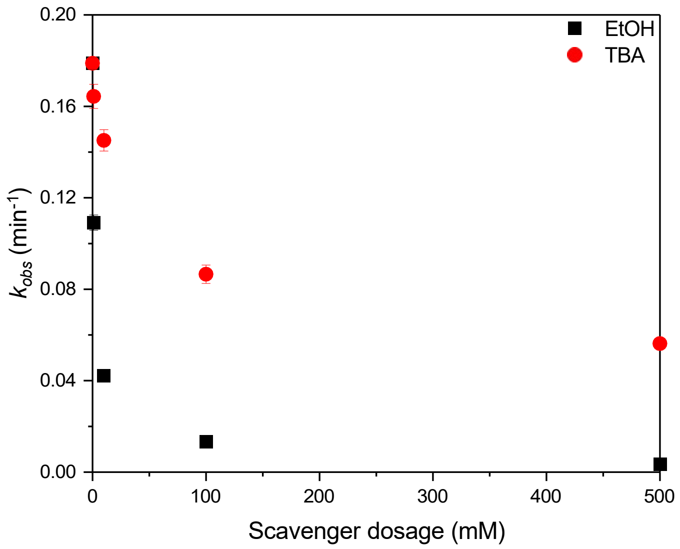

Figure S7. Effect of radical scavenger concentration on AO7 degradation rate constants (k_{obs}) in the Fe(III)/MBS process (Reaction condition: $[AO7]_0$ = 0.02 mM, $[Fe(III)]_0$ = 0.01 mM, $[MBS]_0$ = 0.1 mM, T = 25 °C).

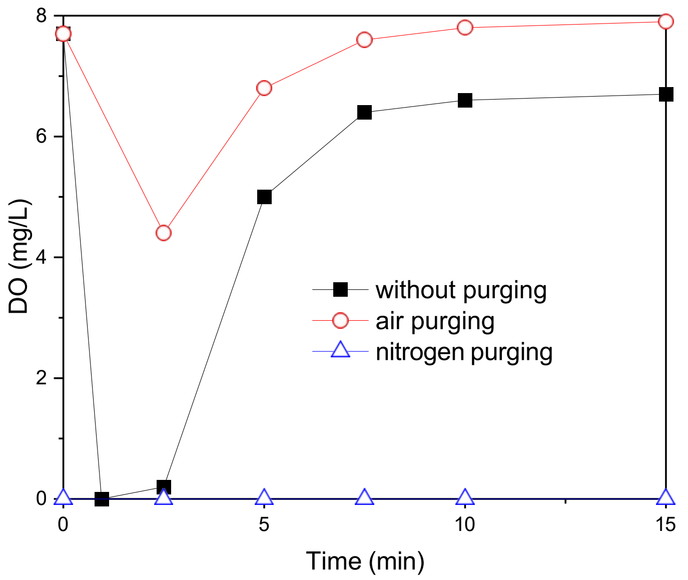

Figure S8. Evolution of dissolved oxygen during AO7 degradation in the Fe(III)/MBS process (Reaction condition: $[AO7]_0 = 0.02$ mM, $[Fe(III)]_0 = 0.01$ mM, $[MBS]_0 = 0.1$ mM, T = 25 °C).

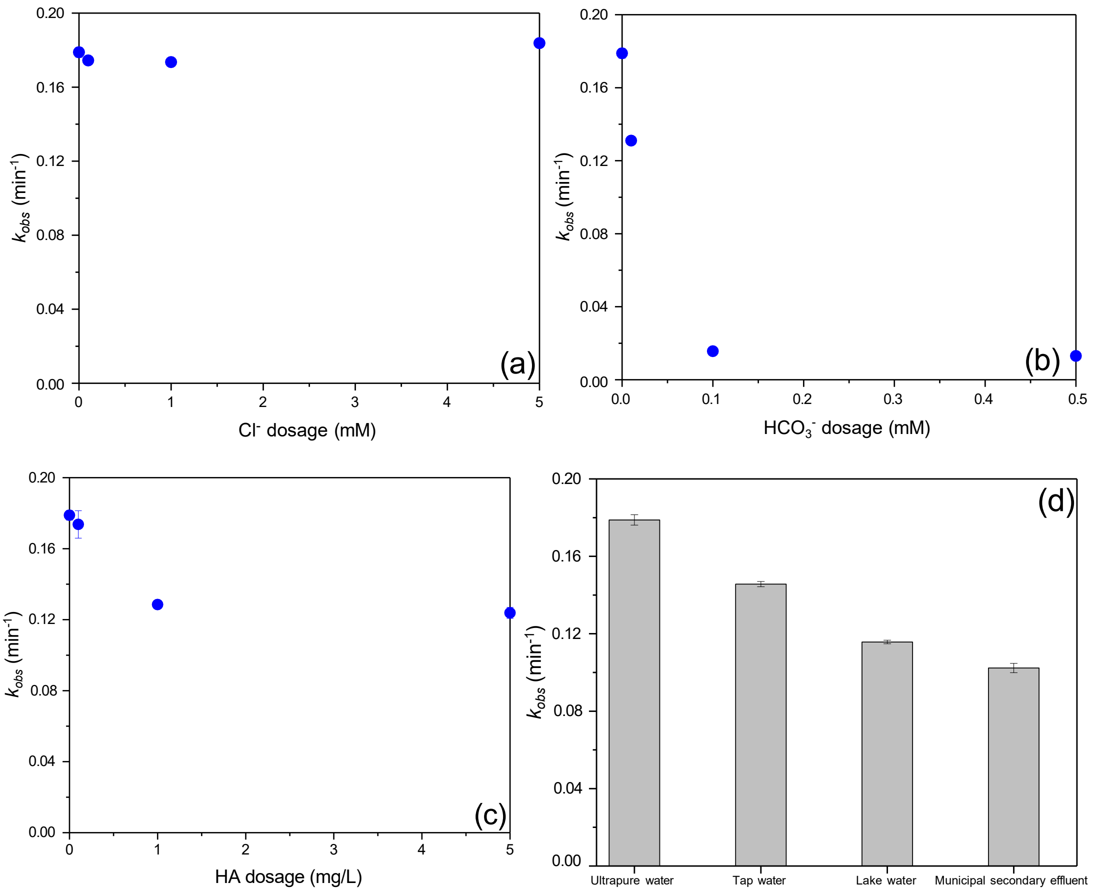

Figure S9. Effect of initial (a) Cl^-, (b) HCO_3^-, (c) HA concentration and (d) real water matrix on AO7 degradation rate constants (k_{obs}) in the Fe(III)/MBS process (Reaction condition: $[AO7]_0$ = 0.02 mM, $[Fe(III)]_0$ = 0.01 mM, $[MBS]_0$ = 0.1 mM, T = 25 °C).

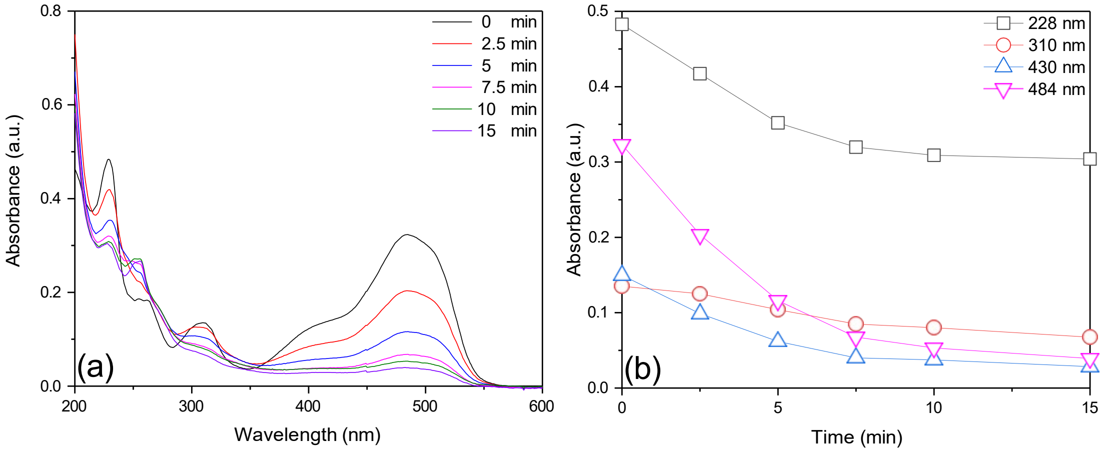

Figure S10. (a) Changes in UV–vis spectrum and (b) UV484, UV430, UV310, and UV228 values of AO7 solutions treated in the Fe(III)/MBS process (Reaction condition: $[AO7]_0 = 0.02$ mM, $[Fe(III)]_0 = 0.01$ mM, $[MBS]_0 = 0.1$ mM, T = 25 °C).

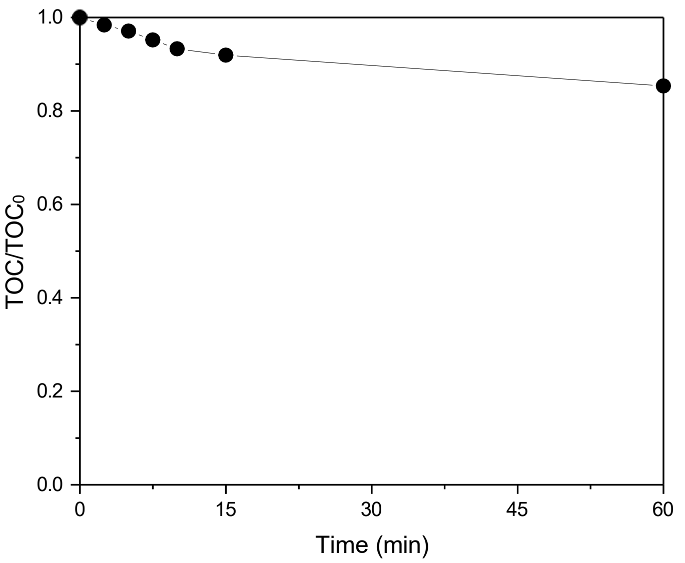

Figure S11. Mineralization of AO7 during Fe(III)/MBS treatment (Reaction condition: $[AO7]_0$ = 0.02 mM, $[Fe(III)]_0$ = 0.01 mM, $[MBS]_0$ = 0.1 mM, T = 25 °C).

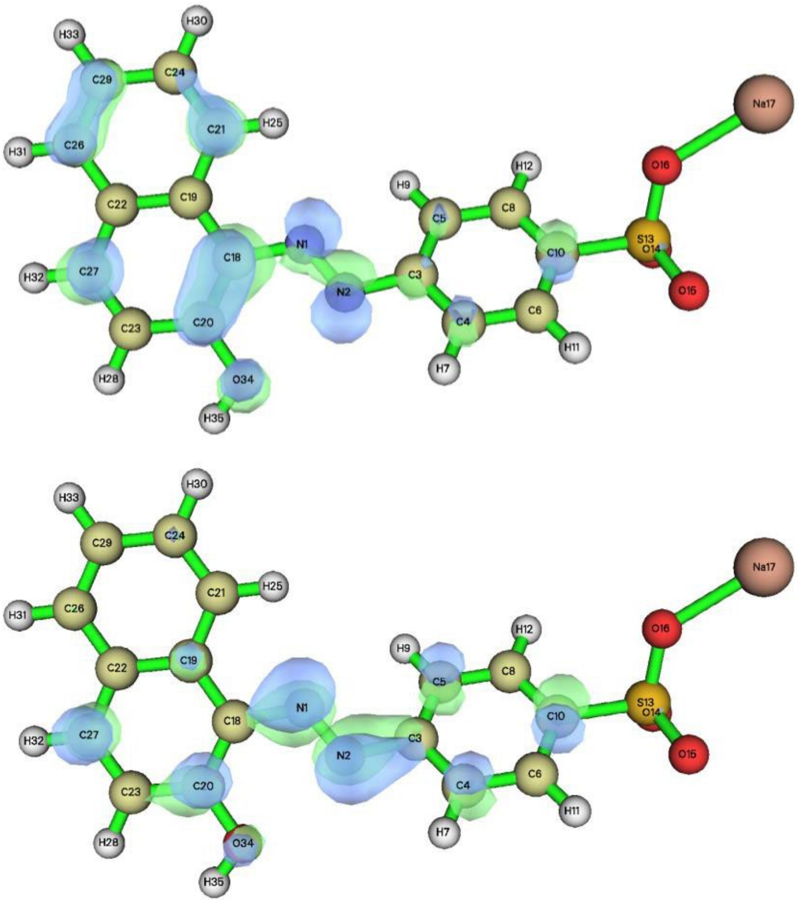

Figure S12. The (top) HOMO and (bottom) LUMO orbits of the AO7 molecule. (The contents of this figure are not legible. Please replace the image with one of a sufficiently high resolution (min. 1000 pixels width/height, or a resolution of 300 dpi or higher).)

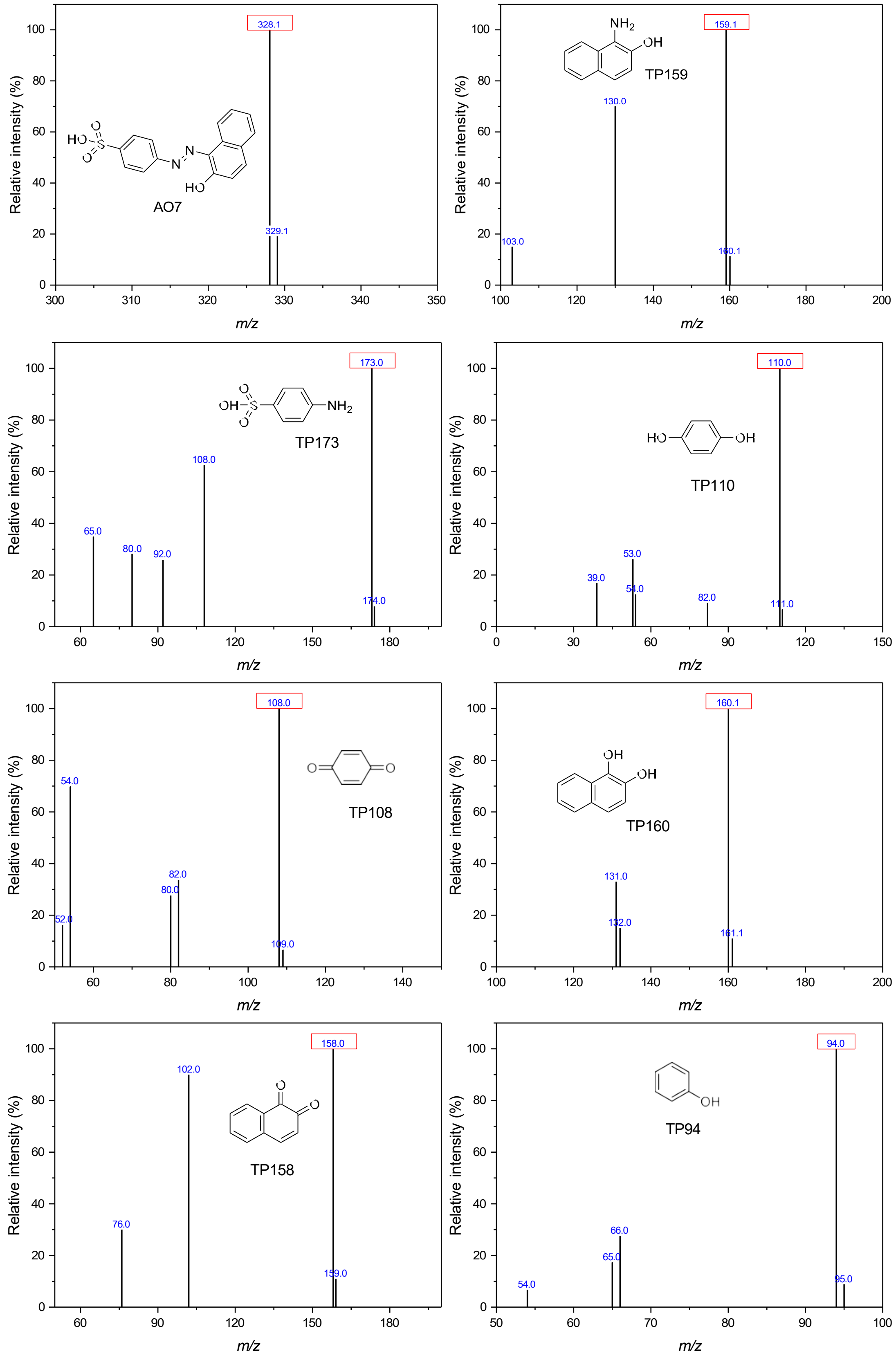

Figure S13. Mass spectrum of the transformation products of AO7 degradation in the Fe(III)/MBS process.

References

1. Zhou, D.; Yuan, Y.; Yang, S.; Gao, H.; Chen, L., Roles of oxysulfur radicals in the oxidation of acid orange 7 in the Fe(III)–sulfite system. *Journal of Sulfur Chemistry* **2015,** 36, (4), 373-384.
2. Yu, Y.; Li, S.; Peng, X.; Yang, S.; Zhu, Y.; Chen, L.; Wu, F.; Mailhot, G., Efficient oxidation of bisphenol A with oxysulfur radicals generated by iron-catalyzed autoxidation of sulfite at circumneutral pH under UV irradiation. *Environmental Chemistry Letters* **2016,** 14, (4), 527-532.
3. Yuan, Y.; Luo, T.; Xu, J.; Li, J.; Wu, F.; Brigante, M.; Mailhot, G., Enhanced oxidation of aniline using Fe(III)-S(IV) system: Role of different oxysulfur radicals. *Chem. Eng. J.* **2019,** 362, 183-189.
4. Dong, H.; Wei, G.; Yin, D.; Guan, X., Mechanistic insight into the generation of reactive oxygen species in sulfite activation with Fe(III) for contaminants degradation. *Journal of Hazardous Materials* **2020,** 384, 121497.
5. Wang, S.; Wang, G.; Fu, Y.; Wang, H.; Liu, Y., A simple Fe3+/bisulfite system for rapid degradation of sulfamethoxazole. *RSC Advances* **2020,** 10, (50), 30162-30168.
6. Xie, P.; Zhang, L.; Wang, J.; Zou, Y.; Wang, S.; Yue, S.; Wang, Z.; Ma, J., Transformation of tetrabromobisphenol a in the iron ions-catalyzed auto-oxidation of HSO32−/SO32− process. *Separation and Purification Technology* **2020,** 235, 116197.
7. Gao, Y.; Fan, W.; Zhang, Z.; Zhou, Y.; Zeng, Z.; Yan, K.; Ma, J.; Hanna, K., Transformation mechanisms of iopamidol by iron/sulfite systems: Involvement of multiple reactive species and efficiency in real water. *Journal of Hazardous Materials* **2022,** 426, 128114.
8. Liu, T.; Xie, Z.; Zhou, P.; Xiong, Z.; Zhang, H.; Pan, Z.; Liu, Y.; Lai, B., Enhanced degradation of carbamazepine by iron/S(IV) system using a novel S(IV) source. *Chem. Eng. J.* **2022,** 431, 133464.
9. Wang, Z.; Bai, F.; Cao, L.; Yue, S.; Wang, J.; Wang, S.; Ma, J.; Xie, P., Activation of sulfite by ferric ion for the degradation of 2,4,6-tribromophenol with the addition of sulfite in batches. *Chinese Chemical Letters* **2022,** 33, (11), 4766-4770.
10. Wang, C.; Huo, Y.; Lu, W.; Shen, X.; Xu, L., A comparative study of sulfite activation using different transition metal ions for the degradation of bisphenol A. *Journal of Environmental Chemical Engineering* **2024,** 12, (2), 112432.
